# Identification and follow up of cardiovascular disease risk factors among participants at a pharmacy student-led screening program

**DOI:** 10.1016/j.rcsop.2025.100636

**Published:** 2025-07-22

**Authors:** Umara Bibi Qureshi, Dineo Mpanya, Razeeya Khan, Muhammed Vally, Ané Orchard

**Affiliations:** a Division of Clinical Pharmacy, Department of Pharmacy and Pharmacology, School of Therapeutic Sciences, University of the Witwatersrand, Johannesburg, South Africa; b Division of Cardiology, Department of Internal Medicine, School of Clinical Medicine, Faculty of Health Sciences, University of the Witwatersrand, Johannesburg, South Africa

**Keywords:** Cardiovascular disease, Pharmacy student, Student-led screening, Non-communicable diseases, Risk factors, Screening

## Abstract

**Background:**

The increased prevalence of cardiovascular disease (CVD) and associated risk factors like hypertension, type 2 diabetes, dyslipideamia, and obesity underscores the need for proactive screening. Given the insidious progression of these conditions, early detection is paramount. The Screening and Testing Programme for Pharmacy Students (STEPPS) is a pharmacy student-led, work-based learning initiative at the University of the Witwatersrand that provides preventive cardiovascular risk screening to university staff and students.

**Aim:**

To identify the occurrence of underlying and uncontrolled risk factors for cardiovascular disease from a convenience sample of participants who attended the STEPPS screening events at the University of the Witwatersrand in year 2022. The study further determined whether the referral of the identified participants led to a diagnosis and intervention.

**Methodology:**

A cross-sectional study was conducted in a screening event called STEPPS at the University of the Witwatersrand. A convenience sample of university staff and students aged 18 years and older who voluntarily participated was included. Fourth-year pharmacy students conducted screenings, including blood pressure, blood glucose, cholesterol, and anthropometric measurements. Participants with abnormal results were referred for further care, and follow-up was conducted via telephone interviews several months later. Quantitative data were analysed using descriptive and inferential statistics in STATA® 18.0.

**Results:**

There was a self-reported occurrence of hypertension (6.5 %), diabetes (2.09 %), dyslipideamia (2.87 %), and obesity (3.91 %). Elevated readings were observed among 136 (18.25 %) participants for blood pressure, 13 (2.83 %) participants for blood glucose and, 50 (11.36 %) participants for blood cholesterol. Among the CVD-related referrals based on abnormal screening results (33 participants), 75 % complied. Of these, 35 % exhibited significant findings, including newly diagnosed cases (43 %), disease escalation (29 %) and lifestyle modifications (29 %). Among follow-up participants, 16 (80 %) participants reported undergoing interventions post-screening.

**Conclusion:**

The student-led initiative effectively identified the occurrences of undiagnosed and uncontrolled cases at the university with 80 % of referrals leading to a medical intervention.

## 1 Introduction

Non-communicable diseases (NCDs), including cardiovascular diseases (CVDs) and diabetes, are the leading global causes of morbidity and mortality, accounting for 74 % of deaths.^2^, ^3^, 4. Most of these occur in low- and middle-income countries (LMICs)^4^ such as South Africa, where poverty and adverse socioeconomic conditions contribute to earlier onset and poor health prognosis compared to more developed nations.^2^^,^^5^^,^^6^ South Africa faces a quadruple disease burden, with CVDs constituting a significant challenge due to their asymptomatic progression and delayed detection.^2^^,^^7^ Screening enables the early identification and staging of diseases during their presymptomatic phase, making it essential to offer screening and monitoring services to asymptomatic individuals.^8^, ^9^, ^10^ A health-enabling environment facilitates the opportunity for asymptomatic individuals to screen themselves for CVD risk.^9^^,^^10^

There is a broad consensus that preventing CVDs is more cost-effective and beneficial to public health than treating complications after onset. Non-communicable diseases (NCDs), including CVDs, are largely preventable through targeting modifiable lifestyle risk factors such as physical inactivity, unhealthy diets, and harmful alcohol and tobacco use.^11^ The increasing morbidity and mortality due to NCDs strain the delivery of health services, diminish quality of life, and negatively impact the economy.^7^^,^^11^ Approximately ZAR 2.7 billion (USD 146 million) was spent by the public sector in 2018 on diabetes patients, with total costs including undiagnosed diabetes cases reaching ZAR 21.8 billion (USD 1.1 billion).^12^

Early detection of NCDs and NCD risk factors enables timely lifestyle modifications and/or initiated pharmacological treatment to manage the condition^13^^,^^14^ to prevent end-organ damage resulting in morbidity and premature mortality.^15^^,^^16^ National initiatives, such as Mayosi et al*'s*^7^ advocation for government-academic collaboration is comparable to Hofman's^15^ proposal of a Health Promotion Foundation (HPF) to raise awareness and monitor NCD trends. As there are healthcare inequalities and the healthcare system is already overburdened, task-sharing initiatives have been recommended, including the involvement of medical students to conduct screening activities.^16^^,^^17^ Banack et al.^14^ highlights that pharmacists often lack the time to provide health promotion and CVD preventive care services. In comparison, student-led initiatives offer both valuable clinical training/practical learning opportunities and improved access to healthcare for underserved communities.^14^^,^^17^^,^^18^

Internationally, pharmacy schools integrate work-based learning (WBL) and community outreach events to strengthen clinical competencies.^9^^,^^19^ This sentiment is shared by pharmacy students as there are insufficient experiential learning opportunities available to practice clinical skills.^20^ Health promotion and screening initiatives providing Point of Care Testing (POCT) allow the students to practice clinical skills in real-life controlled environments under the supervision of facilitators with minimal risk to the patients.^19^^,^^21^ Health promotion and screening initiatives should be hosted at universities, workplaces and extend to community outreach events/public spaces where the public frequents for daily tasks to make screening more accessible. Furthermore, Yamamoto-Moreno *et al*^22^ highlight how these events allow the opportunity for those people who don't perform regular health screening, or have limited access, an opportunity to test CVD biochemical markers. Essentially, it is the student-led initiatives outside the hospital that facilitate access to pathways to care.^10^ Universities and workplaces are ideal settings for health promotion and prevention efforts as individuals from diverse socio-economic and cultural backgrounds spend considerable time in these places.^23^, ^24^, ^25^, ^26^ University students tend to adopt sedentary, unhealthy lifestyles due to academic pressures,^26^^,^^27^ while the working population commonly exhibit risky behaviours and multiple risk factors for NCDs.^23^ Promoting the health and well-being of individuals in academic and workplace settings can enhance student performance and employee productivity, offering a strategic opportunity to influence risk behaviour and promote the health of the population.^23^^,^^24^^,^^27^

Considering the benefit of reaching previously overlooked communities, the clinical pharmacy division from the Department of pharmacy and pharmacology at the University of the Witwatersrand launched a preventative health screening and education programme as part of the work-based learning initiative for pharmacy students called Screening and Testing Programme for Pharmacy Students (STEPPS). Fourth-year pharmacy students played a central role in the implementation of the screening initiative. Prior to participation, pharmacy students undergo intensive training on the use of screening equipment and the procedures for obtaining key cardiovascular biomarkers, such as blood pressure, blood glucose, total cholesterol, and body mass index (BMI). Competency is assessed through a summative theory examination and a formative Objective Structured Clinical Examination (FOSCE) before students are allowed to participate. Any student deemed as not yet ready undergoes continuous revision and retesting before joining the STEPPS events. This study aimed to identify the occurrence of underlying or uncontrolled cardiovascular diseases from a convenience sample of participants who attended the STEPPS screening events at the University of the Witwatersrand for the year 2022. The study further determined whether the referral of the identified patients led to a diagnosis and intervention.

## 2 Materials and methods

### 2.1 Study design, study population and study setting

A cross-sectional study was conducted at the University of the Witwatersrand, encompassing a total of 911 participants, including both university staff and students, who were screened during the STEPPS events held throughout 2022. The STEPPS initiative is also open to any community member that may be visiting or brought by a staff member or student. The university was selected as the study site due to its accessibility, the availability of a large venue suitable for such events, and its alignment with budgetary constraints. Moreover, the population was deemed appropriate given the predominance of students, many of whom face financial barriers to accessing routine health screening services.

A comprehensive review of the STEPPS 2022 dataset was undertaken to ensure the integrity of the analysis. Records were excluded if they were incomplete, contained unreliable or erroneous readings, or lacked gender disclosure, particularly to preserve the validity of gender-based health assessments. Following this data refinement, a convenience sample of 767 individuals aged 18 years and older was included in the final analysis. Convenience sampling was employed due to logistical considerations and the practical advantages of recruiting participants within the university setting. Additionally, follow-up surveys were administered to individuals who had been referred for cardiovascular risk factors, facilitating further evaluation and potential intervention.

### 2.2 Biochemical markers, anthropometric measurements, and demographics

Each participant was screened for the presence of risk factors using a structured questionnaire (S1), which captured medical history and demographic data. The questionnaire was completed with the assistance of a trained student. Participants self-reported the presence of hypertension, diabetes, dyslipidaemia, and obesity. Blood pressure (BP) was measured three times, and the average of these readings was used for analysis. Hypertension was defined as a systolic blood pressure (SBP) ≥ 140 mmHg and/or a diastolic blood pressure (DBP) ≥ 90 mmHg. Blood glucose levels were assessed using either fasting blood glucose (FBG) or random blood glucose (RBG) measurements. Abnormal glucose levels were defined as FBG ≥ 5.6 mmol/L or RBG ≥ 11.1 mmol/L.^28^ Total cholesterol (TC) levels ≥5.0 mmol/L were considered indicative of dyslipidaemia. Participants presenting with abnormal FBG, RBG, or TC values or with a relevant medical history were offered further testing, including HbA1c and a full lipogram. Those with abnormal findings were referred for further evaluation to the university medical doctor, a primary care clinic, or their preferred healthcare provider. Anthropometric measurements were taken to calculate Body Mass Index (BMI), with values ≥30.0 kg/m^2^ classified as obese. Waist circumference (WC) was also measured, with thresholds of ≥94 cm for men and ≥ 80 cm for women used to define central (abdominal) obesity and elevated risk for NCDs.^28^^,^^29^

### 2.3 Follow-up surveys

**Fig. 1:**
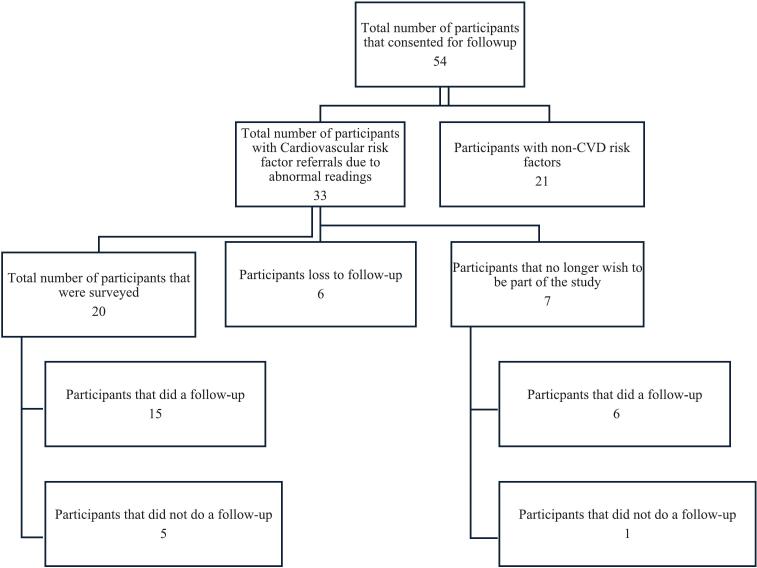
STEPPS CVD referral sample selection.

A follow-up survey was conducted with twenty consenting participants (whom had granted permission to be followed up on) via telephone, using a structured questionnaire (S2). Informed consent was obtained from all participants prior to the interviews. The selection process for follow-up participants is illustrated in Fig. 1. The telephonic questionnaire primarily comprised closed-ended questions and was designed to gather comprehensive data aligned with the study objectives. This included demographic information, current health status, details of any health interventions undertaken following the STEPPS screening event, and participant feedback regarding the screening experience.

### 2.4 Statistical analysis

Data collection was managed using REDCap® version 14.1.4. Upon completion, the dataset was exported, cleaned, and securely stored in Microsoft Excel Office® 365 on a password-protected computer. Statistical analyses were performed using STATA/SE® version 18.0. Biochemical markers were classified as either normal or abnormal based on clinical thresholds outlined in Supplementary Table S3. These classifications were compared across demographic variables using appropriate statistical tests, including ANOVA, *t*-tests, and the Kruskal-Wallis test, depending on data distribution. Statistical significance was set at *p* < 0.05. Where applicable, post hoc analyses were conducted using Bonferroni's and Dunn's tests to further explore significant group differences.

### 2.5 Ethics and consent

Ethics approval was obtained from the Human Research Ethics Committee (Medical) of University of the Witwatersrand (M220918) and consent was obtained from each participant to conduct the study.

## 3 Results

### 3.1 Study population

A total of 767 university staff and students were included in the study (Table 1). Of these, 511 (66.62 %) identified as female, and 544 (70.93 %) were students. Many of the participants were between the ages of 18 and 24, with 633 (82.53 %) of African origin or ethnicity. Among the 734 respondents to the screening behaviour questions, 348 (47.41 %) indicated that they only undergo health screening when it is offered as part of an organized event. Additionally, 330 (44.96 %) reported that the STEPPS screening was their first experience participating in such an initiative.

**Table 1:** Socio-demographic characteristics and family history of participants.

		*N* = 767 (Freq.)	Percent %
**Gender**	Female	511	66.62
	Male	235	30.64
	Unknown	21	2.74
	**Total**	767	100

**Age range**	18–24	512	66.75
	25–34	96	12.52
	35–44	66	8.6
	45–54	55	7.17
	55–64	26	3.39
	65 and above	3	0.39
	Unknown	9	1.17
	**Total**	767	100

**Ethnicity**	African	633	82.53
	Asian	29	3.78
	Indian	53	6.91
	Mixed Ancestry	21	2.74
	White	23	3
	Unknown	8	1.04
	**Total**	767	100

**Designation**	Staff	180	23.47
	Students	544	70.93
	Other	11	1.43
	Unknown	32	4.17
	**Total**	767	100

**Medical aid**	Yes	286	37.29
	No	457	59.58
	Unknown	24	3.13
	**Total**	767	100

**Positive family history**	Hypertension	276	35.98
(self-reported)	Diabetes	275	35.85
	Cholesterol	115	14.99
	Obesity	69	9

**Alcohol Consumption**	1–3 times per month	252	32.86
	1–6 times per week	42	5.48
	Daily	2	0.26
	Never or rarely	464	60.5
	Unknown	7	0.91
	**Total**	767	100

**Smoking**	No	574	74.84
	No, but I used to	52	6.78
	Only socially	78	10.17
	Yes	52	6.78
	Unknown	11	1.43
	**Total**	767	100

### 3.2 Biochemical markers, anthropometric measurements, and demographics

The results of the screening tests are summarized in Table 2. Elevated blood pressure readings were observed in 136 participants (18.25 %). Hyperglycaemia was detected in 13 participants (2.83 %), with 2.17 % identified through fasting blood glucose and 0.65 % through random blood glucose measurements. Of those with hyperglycaemia, three underwent HbA1c testing, and two were found to have HbA1c levels ≥6.5 %, indicating possible diabetes.

**Table 2:** Screening results.

Screening results and measurements	Frequency (n)	Percentage (%)
***Blood pressure readings***		
Normal (<130 mmHG systolic and < 85 mmHG diastolic)	294	38.33
High Normal (130–139 mmHG systolic and 85–89 mmHG diastolic)	315	41.07
Isolated Diastolic (< 140 mmHG systolic and ≥ 90 mmHG diastolic)	84	10.95
Isolated Systolic (≥ 140 mmHG systolic and < 90 mmHG diastolic)	16	2.09
Elevated blood pressure reading (≥ 140 mmHG systolic and ≥ 90 mmHG diastolic)	36	4.69
Not measured	22	2.87
***Total***	***767***	***100***

***Blood Glucose readings***		
Random Hypoglycaemia (<4.0 mmol/L)	84	10.95
Random Normal (5.6–11.0 mmol/L)	306	39.9
Random Hyperglycaemia (≥11.1 mmol/L)	3	0.39
Fasting Hypoglycaemia (<4.0 mmol/L)	17	2.22
Fasting Normal (< 5.6 mmol/L)	40	5.22
Fasting Hyperglycaemia (≥ 5.6 mmol/L)	10	1.3
Not measured	307	40.03
***Total***	***767***	***100***

***Blood Cholesterol readings***		
High Cholesterol (TC ≥ 5.0 mmol/L)	50	6.52
Normal Cholesterol (TC <4.9 mmol/L)	390	50.85
Not tested	327	42.63
***Total***	***767***	***100***

**Body Mass Index (BMI)**		
Underweight (<18.5 kg/m^2^)	37	4.69
Normal weight (<18.5 kg/m^2^–24.9 kg/m^2^)	230	29.07
Overweight (25.0 kg/m^2^–29.9 kg/m^2^)	134	16.95
Obese (≥ 30.0 kg/m^2^)	100	12.39
Not measured	266	36.9
***Total***	***767***	***100***

***Waist circumference***		
Increased risk Females (≥ 80 cm)	138	17.99
Increased risk Males (≥ 94 cm)	34	4.43
Normal Waist Circumference Females (< 80 cm)	212	27.64
Normal Waist Circumference Males (< 94 cm)	108	14.08
Not measured	275	35.85
***Total***	***767***	***100***

High total cholesterol levels were recorded in 50 participants (11.36 %). Among those who underwent further lipid panel testing, six had elevated low-density lipoprotein cholesterol (LDL-C ≥ 3.0 mmol/L), four had elevated triglycerides (TG ≥ 1.7 mmol/L), and one had high high-density lipoprotein cholesterol (HDL—C). Although eligible participants were offered comprehensive lipid profiling, incomplete data entry resulted in missing values for HDL—C, LDL-C, and TG in some cases.

Among the screened participants, 50 (6.50 %) self-reported a history of hypertension, 16 (2.09 %) reported diabetes, 22 (2.87 %) reported dyslipidaemia, and 30 (3.91 %) reported obesity. Of those with self-reported hypertension (Table 3), 28 (56.00 %) exhibited uncontrolled blood pressure readings. Among participants with diabetes, three (18.75 %) presented with hyperglycaemia. In the dyslipidaemia group, four (18.18 %) had elevated total cholesterol levels. Of the individuals reporting obesity, 15 (50.00 %) were confirmed to meet the clinical criteria for obesity. In addition, 105 participants (15.51 %) without a prior diagnosis of hypertension displayed elevated blood pressure readings (systolic ≥140 mmHg and/or diastolic ≥90 mmHg). Among those without a history of diabetes, nine (2.05 %) had fasting blood glucose levels exceeding 5.6 mmol/L. Elevated total cholesterol levels were observed in 45 (10.74 %) participants without a known history of dyslipidaemia. Furthermore, among participants who did not self-identify as obese, 79 (16.88 %) were found to meet the criteria for obesity, and an additional 128 (27.35 %) were classified as overweight.

**Table 3:** Screening results and diagnosis of NCD.

Results of the screening tests and measurements*	History of Hypertension						History of Diabetes						History of Cholesterol						History of Obesity					
	No		Yes		Total		No		Yes		Total		No		Yes		Total		No		Yes		Total	
	N	%	N	%	N	%	N	%	N	%	N	%	N	%	N	%	N	%	N	%	N	%	N	%
*Blood Pressure readings*																								
Normal (<130 mmHG systolic and < 85 mmHG diastolic)	283	41.8	3	6	286	39.3	283	39.9	3	18.8	286	39.4	286	40.6	3	13.6	289	39.8	278	40.2	7	24.1	285	39.6
High Normal (130–139 mmHG systolic and 85–89 mmHG diastolic)	289	42.7	19	38	308	42.4	303	42.7	5	31.2	308	42.4	296	42	10	45.5	306	42.1	298	43.1	8	27.6	306	42.5
Isolated Diastolic (< 140 mmHG systolic and ≥ 90 mmHG diastolic)	70	10.3	13	26	83	11.4	81	11.4	3	18.8	84	11.6	82	11.6	1	4.5	83	11.4	75	10.9	8	27.6	83	11.5
Isolated Systolic (≥ 140 mmHG systolic and < 90 mmHG diastolic)	12	1.8	4	8	16	2.2	14	2	2	12.5	16	2.2	13	1.8	3	13.6	16	2.2	15	2.2	1	3.4	16	2.2
Elevated BP (≥ 140 mmHG systolic and ≥ 90 mmHG diastolic)	23	3.4	11	22	34	4.7	29	4.1	3	18.8	32	4.4	28	4	5	22.7	33	4.5	25	3.6	5	17.2	30	4.2
Total	677	100	50	100	727	100	710	100	16	100	726	100	705	100	22	100	727	100	691	100	29	100	720	100

*Blood Glucose readings*																								
Random Hypoglycaemia (<4.0 mmol/L)	81	11.7	1	2	82	11	82	11.3	0	0	82	11	81	11.2	1	4.5	82	11	79	11.2	3	10	82	11.1
Random Normal (5.6–11.0 mmol/L)	279	40.2	23	46	302	40.6	294	40.4	8	50	302	40.6	292	40.4	8	36.4	300	40.3	284	40.1	14	46.7	298	40.4
Random Hyperglycaemia (≥11.1 mmol/L)	1	0.1	1	2	2	0.3	0	0	3	18.8	3	0.4	0	0	2	9.1	2	0.3	2	0.3	0	0	2	0.3
Fasting Hypoglycaemia (<4.0 mmol/L)	15	2.2	2	4	17	2.3	17	2.3	0	0	17	2.3	17	2.4	0	0	17	2.3	15	2.1	2	6.7	17	2.3
Fasting Normal (< 5.6 mmol/L)	38	5.5	2	4	40	5.4	38	5.2	1	6.2	39	5.2	39	5.4	1	4.5	40	5.4	38	5.4	1	3.3	39	5.3
Fasting Hyperglycaemia (≥ 5.6 mmol/L)	7	1	2	4	9	1.2	9	1.2	0	0	9	1.2	9	1.2	0	0	9	1.2	9	1.3	0	0	9	1.2
Not tested	273	39.3	19	38	292	39.2	288	39.6	4	25	292	39.2	285	39.4	10	45.5	295	39.6	281	39.7	10	33.3	291	39.4
Total	694	100	50	100	744	100	728	100	16	100	744	100	723	100	22	100	745	100	708	100	30	100	738	100

*Blood Cholesterol readings*																								
High Cholesterol (TC ≥ 5.0 mmol/L)	44	6.3	6	12.2	50	6.7	47	6.5	1	6.2	48	6.5	45	6.2	4	18.2	49	6.6	44	6.2	3	10	47	6.4
Normal Cholesterol (TC <4.9 mmol/L)	360	51.9	24	49	384	51.7	375	51.6	10	62.5	385	51.8	374	51.8	11	50	385	51.7	367	51.9	15	50	382	51.8
Not tested	290	41.8	19	38.8	309	41.6	305	42	5	31.2	310	41.7	303	42	7	31.8	310	41.7	296	41.9	12	40	308	41.8
Total	694	100	49	100	743	100	727	100	16	100	743	100	722	100	22	100	744	100	707	100	30	100	737	100

*Body Mass Index (BMI)*																								
Underweight (<18.5 kg/m^2^)	36	7.73	0	0	36	7.33	35	7.29	1	8.3	36	7.32	36	7.50	0	0	36	7.35	36	7.69	0	0	36	7.39
Normal weight (<18.5 kg/m^2^–24.9 kg/m^2^)	225	48.28	2	8	227	46.23	224	46.67	3	25	227	46.14	226	47.08	2	20	228	46.53	225	48.08	1	5.3	226	46.41
Overweight (25.0 kg/m^2^–29.9 kg/m^2^)	123	26.39	8	32	131	26.68	131	27.29	1	8.3	132	26.83	130	27.08	1	10	131	26.73	128	27.35	3	15.8	131	26.90
Obese (≥ 30.0 kg/m^2^)	82	17.60	15	60	97	19.76	90	18.75	7	58.3	97	19.72	88	18.33	7	70	95	19.39	79	16.88	15	78.9	94	19.30
Total	466	100	25	100	491	100	480	100	12	100	492	100	480	100	10	100	490	100	468	100	19	100	487	100

*Waist circumference*																								
Increased risk females (≥ 80 cm)	118	25.6	16	76.2	134	27.8	130	27.4	3	50	133	27.7	128	27	4	66.7	132	27.5	118	25.4	14	87.5	132	27.5
Increased risk males (≥ 94 cm)	30	6.5	3	14.3	33	6.8	33	6.9	0	0	33	6.9	32	6.8	1	16.7	33	6.9	32	6.9	1	6.2	33	6.9
Normal waist circumference females (< 80 cm)	208	45.1	0	0	208	43.2	205	43.2	3	50	208	43.2	208	43.9	1	16.7	209	43.5	206	44.4	1	6.2	207	43.1
Normal waist circumference males (< 94 cm)	105	22.8	2	9.5	107	22.2	107	22.5	0	0	107	22.2	106	22.4	0	0	106	22.1	108	23.3	0	0	108	22.5
Total	461	100	21	100	482	100	475	100	6	100	481	100	474	100	6	100	480	100	464	100	16	100	480	100

Supplementary material S3 provides the parameters for analysis

Abnormal blood pressure readings were recorded in 51 of the 284 participants who reported alcohol consumption (17.96 %) and in 17 of the 127 participants who reported smoking (13.39 %) (Table 4). Among alcohol consumers, 6 of 296 participants (2.02 %) presented with hyperglycaemia, while four of 130 smokers (3.08 %) exhibited elevated blood glucose levels. Elevated total cholesterol levels were observed in 19 of the 295 alcohol consumers (6.44 %) and in five of the 130 smokers (3.85 %).

**Table 4:** Results of the screening tests and measurements among consumers of tobacco and alcohol.

Results of the screening tests and measurements	Alcohol consumption								Tobacco consumption					
	1–6 times per week		1–3 times per month		Never or rarely		Total		No		Yes		Total	
	N	%	N	%	N	%	N	%	N	%	N	%	N	%
*Blood Pressure readings*														
Normal	15	35.7	94	38.8	184	40.5	293	39.7	226	37.2	62	48.8	288	39.2
High Normal	16	38.1	108	44.6	187	41.2	311	42.1	262	43.2	48	37.8	310	42.2
Isolated Diastolic	8	19	21	8.7	53	11.7	82	11.1	74	12.2	10	7.9	84	11.4
Isolated Systolic	1	2.4	8	3.3	7	1.5	16	2.2	15	2.5	1	0.8	16	2.2
Elevated Blood Pressure reading	2	4.8	11	4.5	23	5.1	36	4.9	30	4.9	6	4.7	36	4.9
Total	42	100	242	100	454	100	738	100	607	100	127	100	734	100

*Blood Glucose readings*														
Random Hypoglycaemia	8	18.2	23	9.1	51	11	82	10.8	69	11	13	10	82	10.8
Random Normal	8	18.2	88	34.9	208	44.8	304	40	270	43.1	31	23.8	301	39.8
Random Hyperglycaemia	0	0	1	0.4	2	0.4	3	0.4	3	0.5	0	0	3	0.4
Fasting Hypoglycaemia	0	0	4	1.6	13	2.8	17	2.2	16	2.6	1	0.8	17	2.2
Fasting Normal	1	2.3	12	4.8	27	5.8	40	5.3	31	5	9	6.9	40	5.3
Fasting Hyperglycaemia	3	6.8	2	0.8	5	1.1	10	1.3	6	1	4	3.1	10	1.3
Not tested	24	54.5	122	48.4	158	34.1	304	40	231	36.9	72	55.4	303	40.1
Total	44	100	252	100	464	100	760	100	626	100	130	100	756	100

*Blood Cholesterol readings*														
High Cholesterol	1	2.3	18	7.2	31	6.7	50	6.6	45	7.2	5	3.8	50	6.6
Normal Cholesterol	21	47.7	115	45.8	253	54.5	389	51.3	327	52.3	59	45.4	386	51.1
Not tested	22	50	118	47	180	38.8	320	42.2	253	40.5	66	50.8	319	42.3
Total	44	100	251	100	464	100	759	100	625	100	130	100	755	100

*Body Mass Index (BMI)*														
Underweight	0	0	13	7.9	24	7.7	37	7.4	31	7.4	6	7.6	37	7.5
Normal weight	15	65.2	59	35.8	155	49.8	229	45.9	182	43.6	46	58.2	228	46.0
Overweight	8	34.8	57	34.5	68	21.9	133	26.7	112	26.9	20	25.3	132	26.6
Obese	0	0	36	21.8	64	20.6	100	20.0	92	22.1	7	8.9	99	20.0
Total	23	100	165	100	311	100	499	100	417	100	79	100	496	100

A statistically significant association was observed between the occurrence of diabetes, elevated cholesterol, and obesity across different ethnic groups, age categories, and participant designations (*p* < 0.05; Table 5). In contrast, the occurrence of hypertension showed a significant association only with age and designation. Additionally, the distribution of obesity was significantly associated with gender.

**Table 5:** Association between diagnosis and demographic factors.

	History of Hypertension							History of Diabetes							History of Cholesterol							History of Obesity						
	No		Yes		Total		*P* value	No		Yes		Total		P value	No		Yes		Total		P value	No		Yes		Total		P value
	N	%	N	%	N	%		N	%	N	%	N	%		N	%	N	%	N	%		N	%	N	%	N	%	
*Gender*																												
							*P* = 0.274							*P* = 0.123							*P* = 0.086							*P* = 0.003⁎
Female	461	68	37	75.5	498	68.5		484	68	13	86.7	497	68.4		481	68	18	85.7	499	68.5		465	67.2	27	93.1	492	68.2	
Male	217	32	12	24.5	229	31.5		228	32	2	13.3	230	31.6		226	32	3	14.3	229	31.5		227	32.8	2	6.9	229	31.8	
Total	678	100	49	100	727	100		712	100	15	100	727	100		707	100	21	100	728	100		692	100	29	100	721	100	

*Age range*																												
							*P* < 0.001⁎							P < 0.001⁎							P < 0.001⁎							P < 0.001⁎
18–24	494	71.8	6	12	500	67.8		497	68.7	5	33.3	502	68		500	69.6	3	14.3	503	68.1		489	69.6	10	33.3	499	68.1	
25–34	89	12.9	6	12	95	12.9		93	12.9	2	13.3	95	12.9		91	12.7	3	14.3	94	12.7		89	12.7	6	20	95	13	
35–44	51	7.4	12	24	63	8.5		64	8.9	1	6.7	65	8.8		62	8.6	3	14.3	65	8.8		58	8.3	5	16.7	63	8.6	
45–54	39	5.7	12	24	51	6.9		48	6.6	1	6.7	49	6.6		45	6.3	4	19	49	6.6		45	6.4	4	13.3	49	6.7	
55–64	12	1.7	14	28	26	3.5		19	2.6	5	33.3	24	3.3		17	2.4	8	38.1	25	3.4		19	2.7	5	16.7	24	3.3	
65 and above	3	0.4	0	0	3	0.4		2	0.3	1	6.7	3	0.4		3	0.4	0	0	3	0.4		3	0.4	0	0	3	0.4	
Total	688	100	50	100	738	100		723	100	15	100	738	100		718	100	21	100	739	100		703	100	30	100	733	100	

*Ethnicity*																												
							*P* = 0.116							*P* = 0.01⁎							P < 0.001⁎							*P* = 0.002⁎
African	580	84.1	35	71.4	615	83.2		607	83.8	10	62.5	617	83.4		606	84.3	11	52.4	617	83.4		592	84.1	22	73.3	614	83.7	
Asian	24	3.5	5	10.2	29	3.9		29	4	0	0	29	3.9		29	4	0	0	29	3.9		29	4.1	0	0	29	4	
Indian	48	7	5	10.2	53	7.2		51	7	2	12.5	53	7.2		51	7.1	1	4.8	52	7		50	7.1	3	10	53	7.2	
Mixed	18	2.6	2	4.1	20	2.7		18	2.5	2	12.5	20	2.7		16	2.2	3	14.3	19	2.6		14	2	4	13.3	18	2.5	
White	20	3.9	2	4.1	22	3		19	2.6	2	12.5	21	2.8		17	2.4	6	28.6	23	3.1		19	2.7	1	3.3	20	2.7	
Total	690	100	49	100	739	100		724	100	16	100	740	100		719	100	21	100	740	100		704	100	30	100	734	100	

*Designation*																												
							P < 0.001⁎							*P* = 0.014⁎							P < 0.001⁎							P < 0.001⁎
Other	9	1.3	1	2.1	10	1.4		10	1.4	0	0	10	1.4		10	1.4	0	0	10	1.4		10	1.5	1	3.6	11	1.5	
Staff	137	20.4	39	83	176	24.5		165	23.4	8	57.0.1	173	24.1		160	22.8	14	82.4	174	24.2		155	22.6	17	60.7	172	24.1	
Student	526	78.3	7	14.9	533	74.1		529	75.1	6	42.9	535	74.5		531	75.7	3	17.6	534	74.4		522	76	10	35.7	532	74.4	
Total	672	100	47	100	719	100		704	100	14	100	718	100		701	100	17	100	718	100		687	100	28	100	715	100	

⁎ P < 0.05, significant association between the variables.

A statistically significant association was observed between gender and several health indicators, including anthropometric measurements, systolic blood pressure, and total cholesterol levels (Table 6). Based on waist circumference (WC) classification, 34.5 % of participants were identified as having abdominal (central) obesity, with a higher prevalence among females (27.9 %) compared to males (6.7 %). Similarly, general obesity was more common in females (23.87 %) than in males (12.03 %). Among participants with both obesity and increased WC, 68 of 77 (88.3 %) met the criteria, with 72.7 % being female and 15.6 % male (Table 7). Additionally, 58 of 107 participants (54.2 %) were classified as overweight with increased WC, comprising 40.2 % females and 14.0 % males. Significant differences in cholesterol levels were found across ethnic groups (*p* = 0.004). Furthermore, across various age categories and participant designations, statistically significant associations were observed in all biochemical markers, as well as in WC and BMI measurements (*p* < 0.05).

**Table 6:** Association between biochemical marker and measurements with demographic characteristics.

Biochemical Marker and measurements	Outcome		Mean/Median	*P*-Value
Weight (kg)	Gender	Male	71 (63–81)	P < 0.001⁎
		Female	64 (54–75)	
		Total	67 (56–78)	
	Age range	18–24	62 (54–72)	*P* = 0.0001⁎
		25-34	74 (62.5–83)	
		35–44	78 (71–90)	
		45–54	80.5 (71–95)	
		55–64	79 (73–90.25)	
		65 and above	54 (46–92)	
		Total	67 (56–78)	
	Ethnicity	African	67 (56–79)	*P* = 0.5010⁎
		Asian	68 (53–77)	
		Indian	59 (50–79)	
		Mixed Ancestry	61.5 (55.5–72)	
		White	63 (53–74)	
		Total	67 (56–78)	
	Designations	Staff	77.5 (70.5–89)	P = 0.0001⁎
		Student	63 (54.25–73)	
		Other	82 (71.5–94)	
		Total	67 (56–77.5)	
Height (m)	Gender	Male	1.7 (1.57–1.69)	P < 0.001⁎
		Female	1.6 (1.56–1.64)	
		Total	1.6 (1.57–1.69)	
	Age range	18–24	1.62 (1.58–1.69)	*P* = 0.6270
		25–34	1.64 (1.56–1.7)	
		35–44	1.64 (1.59–1.71)	
		45–54	1.64 (1.57–1.7)	
		55–64	1.59 (1.56–1.69)	
		65 and above	1.54 (1.47–1.61)	
		Total	1.62 (1.57–1.69)	
	Ethnicity	African	1.62 (1.58–1.69)	*P* = 0.4350
		Asian	1.68 (1.55–1.72)	
		Indian	1.61 (1.56–1.67)	
		Mixed Ancestry	1.57 (1.56–1.66)	
		White	1.62 (1.57–1.66)	
		Total	1.62 (1.57–1.69)	
	Designations	Staff	1.62 (1.57–1.68)	*P* = 0.5822
		Student	1.62 (1.58–1.69)	
		Other	1.69 (1.6–1.75)	
		Total	1.62 (1.57–1.69)	
Waist circumference (cm)	Gender	Male	80 (73–93))	*P* = 0.0002⁎
		Female	75 (68–87)	
		Total	77 (69–89)	
	Age range	18–24	73.5 (67–80)	P < 0.001⁎
		25-34	87 (75–93)	
		35–44	95.5 (81–102)	
		45–54	94 (89–105)	
		55–64	95 (72–110)	
		65 and above		
		Total	77 (69–89)	
	Ethnicity	African	77 (69–89)	0.2390
		Asian	85.5 (75.5–95.5)	
		Indian	77.5 (65–89)	
		Mixed Ancestry	72.75 (71–87)	
		White	73 (70–77)	
		Total	77(69–89)	
	Designations	Staff	93 (83–102)	P = 0.0001⁎
		Student	74 (67–80)	
		Other	91 (82–102)	
		Total	77 (69–88.5)	
Body Mass Index (BMI) (Kg/m^2^)	Gender	Male	24.03 (21.23–27.17)	*P* = 0.0690
		Female	24.8 (21.21–29.71)	
		Total	24.37 (21.22–28.78)	
	Age range	18–24	22.77 (20.42–25.97)	P = 0.0001⁎
		25-34	27.71 (25.06–31.18)	
		35–44	29.55 (26.17–35.59)	
		45–54	30.62 (26.22–36.37)	
		55–64	29.82 (25.5–34.71)	
		65 and above	–	
		Total	24.25 (21.22–28.7)	
	Ethnicity	African	24.57 (21.23–28.95)	*P* = 0.6530
		Asian	23.88 (22.06–26.41)	
		Indian	22.19 (19.83–28.52)	
		Mixed Ancestry	23.73 (22.14–27.86)	
		White	23.05 (22.6–27.55)	
		Total	24.31 (21.23–28.7)	
	Designations	Staff	29.55 (25.91–34.96)	P = 0.0001⁎
		Student	22.86 (20.45–26.43)	
		Other	33.72 (26.29–37.24)	
		Total	24.24 (21.22–28.52)	
Average Systolic Blood pressure (mmHg)	Gender	Male	125.44	P < 0.001⁎
		Female	115.13	
		Total	118.34	
	Age range	18–24	114.96	*P* = 0.0040⁎
		25-34	119.24	
		35–44	129.09	
		45–54	127.22	
		55–64	134.65	
		65 and above	131.5	
		Total	118.37	
	Ethnicity	African	118.26	*P* = 0.5190
		Asian	119.86	
		Indian	116.63	
		Mixed Ancestry	123.38	
		White	118.15	
		Total	118.34	
	Designations	Staff	126.57	*P* = 0.0010⁎
		Student	115.18	
		Other	126.6	
		Total	118.16	
Average Diastolic Blood pressure (mmHg)	Gender	Male	81.7	*P* = 0.0849
		Female	80.33	
		Total	80.76	
	Age range	18–24	78.11	P < 0.001⁎
		25-34	83.44	
		35–44	88.41	
		45–54	87.66	
		55–64	86.72	
		65 and above	85.5	
		Total	80.68	
	Ethnicity	African	80.52	*P* = 0.5700
		Asian	80.19	
		Indian	81.44	
		Mixed Ancestry	83.77	
		White	81.07	
		Total	80.68	
	Designations	Staff	86.52	P < 0.001⁎
		Student	78.46	
		Other	86.91	
		Total	80.57	
Blood Glucose (mmol/l)	Gender	Male	4.8 (4.1–5.5)	*P* = 0.4390
		Female	4.9 (4.2–5.7)	
		Total	4.8 (4.2–5.7)	
	Age range	18–24	4.8 (4.0–5.5)	*P* = 0.0022⁎
		25-34	4.85 (4.4–5.65)	
		35–44	5.35 (4.4–6.15)	
		45–54	5.4 (4.6–5.9)	
		55–64	5.0 (4.4–5.9)	
		65 and above	6.45 (5.1–7.8)	
		Total	4.8 (4.2–5.7)	
	Ethnicity	African	4.8 (2.94–4.44)	*P* = 0.3650
		Asian	5.5 (4.3–5.7)	
		Indian	5.1 (4.6–5.8)	
		Mixed	5.0 (4.2–5.7)	
		White Ancestry	4.9 (4.3–5.7)	
		Total	4.8 (4.2–5.7)	
	Designations	Staff	5.2 (4.5–6)	P = 0.0010⁎
		Student	4.8 (4–5.5)	
		Other	4.6 (4.3–6)	
		Total	4.8 (4.2–5.7)	
Blood Cholesterol (mmol/l)	Gender	Male	3.2 (2.63–4.16)	P < 0.001⁎
		Female	3.92 (3.2–4.6)	
		Total	3.79 (2.3–4.52)	
	Age range	18–24	3.48 (2.89–4.3)	*P* = 0.0001⁎
		25-34	4.1 (3.49–4.8)	
		35–44	4.41 (3.12–5.3)	
		45–54	4.44 (3.68–5.04)	
		55–64	3.87 (3.48–5.02)	
		65 and above	3.96 (2.92–5)	
		Total	3.77 (2.97–4.47)	
	Ethnicity	African	3.65 (2.94–4.44)	P = 0.0040⁎
		Asian	4.68 (4.13–5.24)	
		Indian	4.09 (3.37–4.73)	
		Mixed Ancestry	3.95 (3.05–4.44)	
		White	3.8 (3.35–5.1)	
		Total	3.78 (2.97–4.5)	
	Designations	Staff	4.44 (3.65–5.03)	P = 0.0001⁎
		Student	3.52 (2.93–4.34)	
		Other	4.2 (3.89–4.38)	
		Total	3.79 (2.99–4.52)	

⁎ P < 0.05, significant association between the variables.

**Table 7:** Waist category compared to the Body Mass Index (BMI).

Waist category	Body Mass Index				
	Underweight	Normal weight	Overweight	Obese	Total
Increased risk Females	1	5	43	56	105
Increased risk Males	0	1	15	12	28
Normal WC Females	21	101	29	7	158
Normal WC Males	4	66	20	2	92
**Total**	26	173	107	77	383

### 3.3 Follow-up surveys

**Fig. 2:**
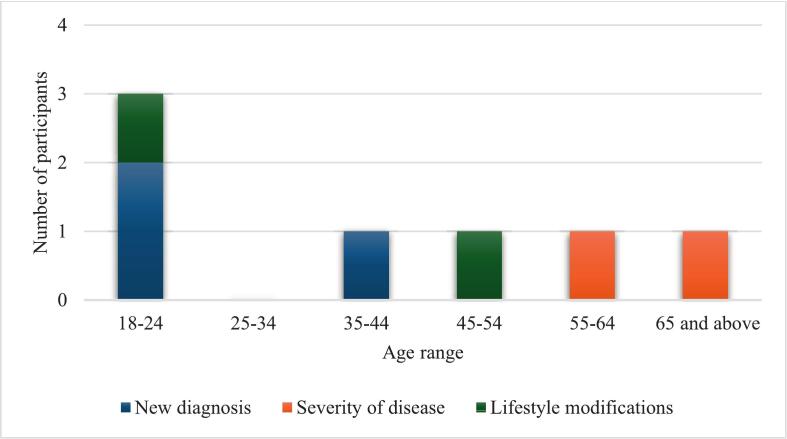
Details of the findings of the referred participants.

**Fig. 3:**
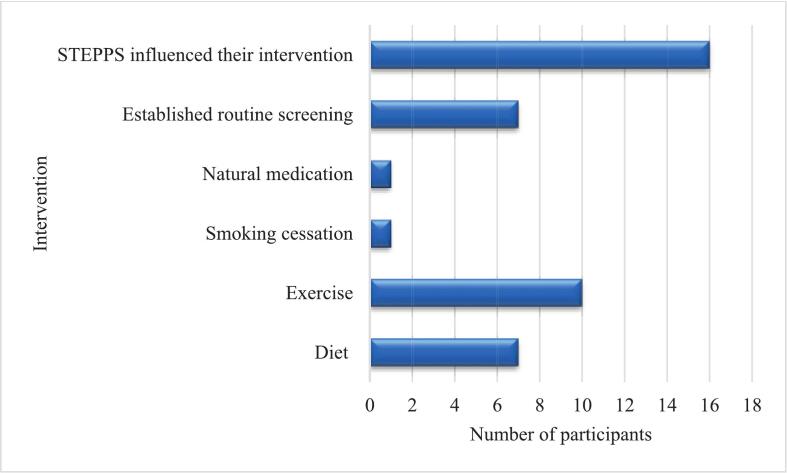
Intervention post-STEPPS screening event.

Of the 33 participants contacted for follow-up, 20 responded, yielding a response rate of 60.61 % (Table 8). Among these, 15 participants (75 %) reported seeking further medical care following the STEPPS screening event. Seven participants disclosed significant outcomes from their follow-up visits (Fig. 2): three received new diagnoses of either hypertension or diabetes, two with pre-existing conditions were informed of disease progression, and two were advised to implement lifestyle modifications to improve their health status. Several participants cited barriers to routine screening, including a lack of perceived need, financial constraints, and time limitations due to academic or work commitments. The health interventions undertaken by participants are illustrated in Fig. 3, with 16 individuals indicating that the STEPPS initiative motivated them to take action. Overall, participant feedback was positive. All 20 respondents expressed comfort with the screening process and affirmed that their results were clearly explained. Furthermore, 75 % (15/20) stated they would “definitely recommend” the service to family and friends, while the remaining 25 % (5/20) indicated they were “likely to recommend” it.

**Table 8:** STEPPS follow-up participants and their responses.

	Frequency (n)	Percent (%)
Age range		
18–24	6	30
25–34	1	5
35–44	4	20
45–54	4	20
55–64	5	25
Total	20	100

Gender		
Female	13	65
Male	7	35
Total	20	100

Further care was sought after STEPPS referral		
No	5	25
Yes	15	75
Total	20	100

Significant findings after further care		
Yes	7	35
No	8	40
Not applicable	5	25
Total	20	100

## 4 Discussion

A cross-sectional study was conducted with a sample of 767 participants at the University of the Witwatersrand, utilizing the STEPPS student-led screening initiative. The STEPPS screening initiative has demonstrated the practicality of empowering pharmacy students to conduct a CVD risk factor screening event at a university. This study highlights a notable occurrence of cardiovascular risk factors among the University staff and students. The results emphasize the importance of routine screening, as a notable proportion of participants who were previously unaware of any conditions exhibited biochemical readings indicative of hypertension, diabetes, dyslipideamia, and obesity. The elevated biochemical readings and measurements may be due to an underlying condition or a pre-diagnosis stage of the condition. As NCDs develop over decades, these participants are unlikely to seek further care until symptomatic.

It is widely acknowledged an individual's demographic profile influences the determination of the CVD risk factor profile.^30^ There are four demographic characteristics, namely the age range, gender, ethnicity, and designation/occupation which were analysed for associations with the occurrence of hypertension, diabetes, cholesterol, obesity, and their biochemical markers. Apart from SBP and DBP, definitive inferences could not be drawn due to the non-normal distribution of the data. The findings of the current study have identified the occurrence of self-reported hypertension as 6.5 %. Among the staff, there were 39 of the 180 (21.67 %) staff members that indicated they have hypertension, this is comparable to the occurrence of hypertension of the staff population at Makerere University, Uganda (20.6 %) and at the University of the Western Cape (UWC), South Africa (26.9 %).^31^^,^^32^ In contrast, there were 7 of the 533 (1.31 %) students that indicated they have hypertension which is comparable the findings at the Walter Sisulu University, where no students where no students were diagnosed with hypertension.^33^ However, much lower than the occurrence of hypertension (22.1 %) among the student population at the King Abdulaziz University (KAU) Jeddah, Kingdom of Saudi Arabia (KSA).^34^ Although all these universities were in the urban region, this discrepancy may be due to the geographical locations, socio-cultural factors, and lifestyles, as Uganda and South Africa may have more similarities as they are both in Africa in comparison to KSA. It may also be due to the availability of healthcare services and as such detection, as KSA is identified as a more developed nation compared to the African countries, which are identified as Low-and-Middle Income Countries (LMIC). Furthermore, it was found that there were 133 of the 727 (18.3 %) participants (14.4 % undiagnosed, 3.8 % previously diagnosed) with high blood pressure readings, indicating the presence of potentially undiagnosed and poorly controlled hypertension in this study. The hypertension occurrence, SBP, and DBP in this study were strongly associated (*P* < 0.05) with the individual's age and designation, indicating that age and the occupation of an individual may influence the BP readings. This was found to be the case in Eng et al^35^ where participants with higher occupational status and more stressful occupations were found to have higher SBP. Consistent with Shisana et al*,*^36^'s findings, there was a significant association between the SBP between the genders (*P* = 0.0000), with the males showing higher SBP means compared to the females. There was no significant association found between the genders for DBP. Although the occurrence of increased blood pressure readings among the males (20.4 %) and females (17.4 %) were similar, the results agree with other studies where the males are more likely to have hypertension compared to females.^31^^,^^37^ Hypertension was also the most common condition reported.

It was found that the occurrence of self-reported diabetes in the current study was 2.09 %. The self-reported occurrence of the staff in this study (4.44 %) and at UWC (5.1 %) were very similar, while the student population at KAU had 15.6 % reported high blood sugar.^32^^,^^34^ The screening results revealed 13 (2.83 %) participants (76.9 % undiagnosed, 23.08 % previously diagnosed) with high blood sugar which was much higher than the undiagnosed population with high blood sugar (45.5 %) found at the upstate New York college.^38^ The authors attribute the high levels of impaired fasting glucose to the high levels of diabetes type 2 risk factors such as physical inactivity (61.4 %) and increased BMI (≥ 25 kg/m^2^) (36.4 %) among the population.^38^ Although the current study revealed that the occurrence of increased BMI was also high (46.7 %), the physical activity level was not assessed. Notably, approximately 36 % of the participants in this study indicated that this was their first screening event which may explain the increased undiagnosed participants. There is limited data on the HbA1c result of the participants, however, among the 13 participants that exhibited high blood sugar, three participants consented to the HbA1c test provided, which revealed two (one was previously diagnosed) of them had high HbA1c levels (≥ 6.5 %). This indicates that one of them was undiagnosed and the other was an uncontrolled diabetic. The presence of undiagnosed diabetes underscores a significant deficiency in regular health check-ups and screenings, potentially leading to severe complications if not promptly addressed. Conversely, the case of uncontrolled diabetes highlights the imperative need for improved management and monitoring of individuals with a diagnosis. Inadequate diabetes control may result from insufficient follow-up, limited access to medications, or a lack of comprehensive patient education on lifestyle modifications and adherence to treatment regimens.

Notably, the age, designation, and ethnicity of participants were significantly associated with the occurrence of diabetes. Both age and designation are also correlated to an individual's blood glucose levels. Tagorda Jr. *et al*^39^ found that age had a significant association with diabetes, with the older population more likely to develop diabetes compared to the younger population. In general, aging was closely linked to cardiovascular and metabolic disorders,^39^^,^^40^ which is consistent with the findings of this study.

Concerning the occurrence of dyslipideamia in this study, the self-reported occurrence of cholesterol was 2.87 %. The staff (7.8 %) had a much lower self-reported cholesterol occurrence compared to the staff at UWC (23.1 %).^32^ This could be due to the staff population at UWC being restricted to administrative staff while this study does not restrict it to admin staff only and may include staff (such as cleaners, academics, researchers and security) that may be more active instead of sedentary compared to staff with administrative roles. The cholesterol screening results indicated that 8.5 % of individuals were potentially undiagnosed or unaware of their cholesterol status. There were 55 participants with one abnormal lipid parameter and at least six participants with two abnormal lipid parameters. It was found that 12.78 % of staff had high cholesterol, although the observed TC was higher than the self-reported cholesterol, indicating high levels of unawareness, however, it was much lower than the measured cholesterol (40 %) of the staff population at a University in KwaZulu Natal.^41^ All demographic characteristics were significantly associated with cholesterol occurrence (*P* < 0.05) while only the blood cholesterol biomarker exhibited a significant association with ethnicity. This is consistent with findings indicating that the ethnicity of an individual may influence the risk of dyslipideamia as it was found that lower TC and higher HDL levels were more common among individuals of African origin compared to other ethnicities.^7^^,^^42^

In this study, it was found that the observed obesity (12.39 %) was three times the self-reported obesity (3.91 %). The screening outcomes among the staff population in this study revealed high rates of classification as overweight (38.8 %) and obesity (44.8 %) which is inconsistent with other studies that had overall lower rates of overweight and obesity.^31^^,^^39^^,^^43^ In contrast, the observation of increased BMI (≥ 25 kg/m^2^) (34.16 %) among the student population was similar to other student populations at the University of Botswana (36.8 %) and New York College (36.4 %).^38^^,^^44^ This could be explained by the demographic difference between the staff and student populations, as most students fall within the 18–24 age range, whereas staff members are generally older. It's commonly observed that as individuals age, they are more likely to experience overweight and obesity.^44^ The occurrence of obesity had a significant association with each of the demographic characteristics (*P* < 0.05), while only certain anthropometric measurements demonstrated a significant association (Table 6). The occurrence of obesity was more prevalent in women (23.87 %) compared to the males (12.03 %) in this study, consistent with the findings of Nakhooda and Wiles,^45^ about the student population of the University of Kwa-Zulu Natal, South Africa. However, international studies have shown a different trend. For instance, McCarthy and Warne^43^ examined gender differences in the physical activity status and knowledge of Irish university students and staff, while Abu Shanab and Al-Sabbah^46^ assessed the prevalence of overweight and obesity among university students at a Palestinian university. Both studies found that the occurrence of obesity was higher among males compared to females.^43^^,^^46^

An individual's BMI is unable to differentiate between lean mass and fat mass and the fat distribution of the individual.^47^ In terms of determining CVD risk, BMI is not the only indicator of risk, central obesity and visceral obesity may also predict CVD risk.^13^^,^^48^ The staff population in this study that was at increased risk (79.4 %) due to abdominal (central) obesity, was comparable to the staff population at the Western Cape University (73.1 %).^32^ In contrast, the student population (21.3 %) with abdominal (central) obesity was found to be lower to a study based at Stellenbosch University that assessed the metabolic risk status among third-year physiology students, where 25 % of female students and 14 % of males exhibited waist circumferences above the accepted range.^49^ This study also found higher occurrences of abdominal obesity among females compared to males. A better indication of CVD risk is the combined occurrence of increased BMI and WC, indicating the presence of android obesity. In the study conducted by Doi and Shah,^47^ obese participants and their anthropometric measurements were compared to their CVD risk factors to compare the risk between gynoid obesity and android obesity. It was found that CVD risk factors were significantly associated with android obesity.^47^ Among those with abdominal obesity, 88.3 % were obese and 54.2 % were overweight.

Among the participants, 130 individuals (16.95 %) reported regular or social smoking. In contrast, a university in Uganda reported no active smokers among its staff, a result attributed to the institution's strict zero-smoking policy and the widespread availability of smoking cessation resources in urban environments.^31^ Notably, a workplace health promotion programme at another university, designed to prevent NCDs, tracked employee health over six years and found that although smokers initially had higher baseline blood pressure than non-smokers, their systolic blood pressure decreased more significantly than that of non-smokers during this period.^35^

Notably, a considerable number (46.9 %) of participants indicated that they engage in screening exclusively when it is offered as a dedicated screening event. One of the reasons cited for the non-establishment of routine screening was the expense involved, however, 37.9 % of the participants indicated that they have medical aid. Interestingly, even among those with medical aid, 45.6 % stated that they only underwent screening during these STEPPS screening events, and ∼ 36 % mentioned that this was their first screening event. This suggests that financial constraints may hinder some participants from organising regular screening, however, others may still choose not to establish routine screening even if it is covered by their medical aid. This highlights the idea that participants are unlikely to actively pursue screening and preventative measures unless they are provided within the framework of their education or work environment. Participants unanimously agreed that the campus was an ideal place to conduct screening and hoped there were more stations and more regular screenings taking place. They further suggested other places where they would like to have similar screening stations, such as malls, community places, and workplaces.

This observation highlights a concerning occurrence of early onset CVD risk factors among young adults. The identification of new cases within this age group underscores the importance of early screening and intervention. Moreover, the provision of lifestyle modification recommendations to a young participant signifies the potential of university-based screening programs to influence health behaviours and potentially mitigate long-term disease progression. These findings suggest that such screening initiatives can play a crucial role in early detection and intervention, thereby potentially reducing the burden of CVD over time. The results align with the research question, which aims to assess the occurrence of cardiovascular diseases identified at the STEPPS screening events during 2022 and to evaluate the impact of a university-based screening programme on the health interventions for referred patients. The data from this study indicates that the screening programme not only identifies individuals at risk but also facilitates timely recommendations and interventions, particularly among younger populations who may benefit the most from early lifestyle changes.

**Fig. 4:**
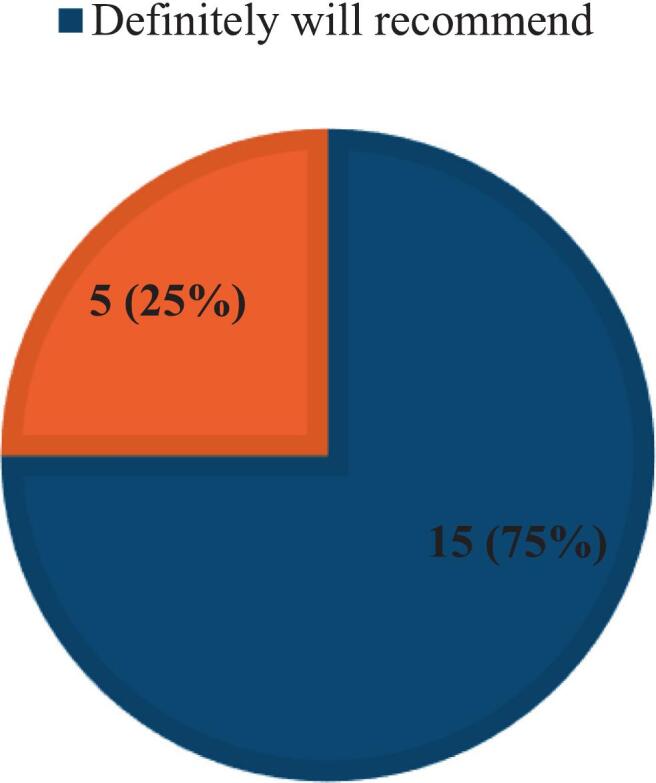
Likelihood of participants recommendation.

Regarding the perception of the participants toward screening services offered, all participants reported that they were comfortable during the screening sessions and the results were explained to them. Furthermore, all participants state that they will “Definitely recommend” (15/20) and are “likely to recommend” (5/20) the screening event to family and friends (Fig. 4). Participants had an overall positive perception regarding pharmacy student-led screening aligning with the conclusions drawn by Bastianelli *et al*^18^ regarding public perception of POCT screening conducted by pharmacy students.

Remarkably, among the reported post-intervention findings, it is noteworthy that the proportion of participants without significant findings (40 %) exceeded the significant ones. This disparity may be attributed to transient factors such as caffeine intake, recent physical activity, or stress that may have influenced the elevated readings during the STEPPS screening. One participant's experience exemplifies this observation, despite being referred for elevated blood pressure readings, subsequent screening revealed normalized blood pressure. The participant attributed the initial elevated reading to heightened academic and personal stress levels at the time of the STEPPS screening event. One participant expressed a preference for student-led screening as there was less fear of health practitioners, and the students were receptive to questions. This suggests that the participant favoured student-led screening over health practitioners' screenings, possibly because they felt less intimidated by the students. It also demonstrates that students were more willing to answer questions, and the participant viewed them as a dependable source of information. This is consistent with the findings observed by Bastianelli *et al*,^18^ which indicate that the patients perceived graduate pharmacy students as a reliable source of general health information.

### 4.1 Limitations

This study faced several limitations that may have influenced the accuracy and generalizability of its findings. Incomplete records and insufficient participant information posed challenges in representing the sample comprehensively, introducing potential biases. As participation was voluntary, the sample may not be representative of the broader university population. Individuals who chose to participate may have been more health-conscious or motivated, potentially skewing the results toward a healthier subset.

The reliance on self-reported data for both health conditions and follow-up behaviours presents a significant limitation, as these responses could not be independently verified. This constraint extends to the assessment of long-term health behaviour changes. Additionally, timing and regularity of follow-up surveys may have introduced recall bias, affecting the reliability of participant responses. Factors such as complex consent procedures and changing contact details, particularly as students graduate or leave the university, contributed to loss to follow-up.

While participant satisfaction was generally high, potential biases must be considered. These include patient bias due to the provision of free healthcare services and social desirability bias, where participants may have overreported positive behaviours or experiences.

Despite the relatively low reported incidence of hypertension, diabetes, dyslipidaemia, and obesity within the cohort, the prevalence of undiagnosed or unrecognized cardiovascular risk factors remains a critical concern. This is underscored by the discrepancy between self-reported and clinically measured cases. Non-modifiable risk factors, such as family history, were notably prevalent among participants. Approximately 36 % reported a family history of hypertension, 35.9 % of diabetes, 15 % of cholesterol issues, and 9 % of obesity, potentially amplifying their overall CVD risk.

Furthermore, modifiable risk factors such as physical activity and dietary intake were not explored in this study, representing a missed opportunity for deeper insight. The voluntary nature of advanced testing (e.g., HbA1c and lipid panels) and follow-up care also introduces the possibility of underestimating the true burden of CVD risk. Therefore, any conclusions regarding the prevalence of these conditions should be interpreted with caution.

### 4.2 Recommendations

A few recommendations may improve this study for future research. To promote the expansion of similar initiatives, other South African universities may consider initiating pharmacy student-led screening and testing programmes. To ensure the completeness of screenings, students should adhere to a more structured approach or employ a therapeutic outline. In addition to the clinical training, students should receive training in data collection techniques. Expanding the data collection tool/questionnaire to include a section on behavioural risk factors could provide valuable insights into the participants' physical activity levels and nutrient intake. Encouraging participants to be more receptive to follow-up surveys is crucial for assessing linkage to care and ensuring participant retention.

## 5 Conclusion

This study evaluated the prevalence of cardiovascular disease (CVD) risk factors among participants of the 2022 STEPPS screening initiative and assessed the effectiveness of a student-led screening model. The findings revealed a notable proportion of participants with abnormal readings for blood pressure, blood glucose, and cholesterol, many of whom were previously undiagnosed or had uncontrolled conditions, highlighting the critical importance of early detection. The STEPPS initiative was well received, with participants reporting high levels of comfort and satisfaction, and many indicating it was their first experience with health screening. These results underscore both the acceptability and public health relevance of such initiatives. Importantly, the STEPPS model demonstrates that pharmacy students can play a meaningful role in conducting health screenings, facilitating early intervention, and contributing to preventative healthcare efforts. Student-led screening models such as this offer a promising approach to bridging gaps in health promotion, particularly in resource-limited settings.

## CRediT authorship contribution statement

**Umara Bibi Qureshi:** Writing – original draft, Methodology, Investigation, Formal analysis. **Dineo Mpanya:** Writing – review & editing, Formal analysis. **Razeeya Khan:** Writing – review & editing, Visualization, Supervision, Methodology, Conceptualization. **Muhammed Vally:** Writing – review & editing, Supervision, Resources, Data curation, Conceptualization. **Ané Orchard:** Writing – review & editing, Visualization, Validation, Supervision, Software, Resources, Project administration, Methodology, Investigation, Funding acquisition, Data curation, Conceptualization.

## Funding

The STEPPS initiative received funding from the South African Department of health Clinical Training Grant, and Aspen Pharmacare in 2022, for the collaboration, consumables and running costs.

## Declaration of competing interest

The authors declare that they have no known competing financial interests or personal relationships that could have appeared to influence the work reported in this paper.

## Data Availability

Permission to use STEPPS 2022 data was granted by the Department of Pharmacy and Pharmacology. Data is available on request.

## Appendix A Supplementary data

Supplementary material Wellness questionairre Supplementary material
