# Supplementary material for: Identification and follow up of cardiovascular disease risk factors among participants at a pharmacy student-led screening program
Source: Explor Res Clin Soc Pharm. 2025 Jul 22;19:100636. doi: 10.1016/j.rcsop.2025.100636 (PMC12329120; doi:10.1016/j.rcsop.2025.100636)
Supplement: Supplementary material — Wellness questionairre [file mmc1.docx]

**Identification and follow-up of participants with Cardiovascular Disease risk factors at a pharmacy-student-led screening and testing programme**

**Authors:**

Umara Bibi Qureshi^1^, Dineo Mpanya^2^, Razeeya Khan^1^, Muhammed Vally^1^, Ané Orchard^1^

**Supplementary materials**

**S1: STEPPS wellness questionnaire**


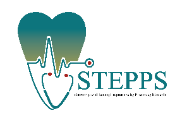

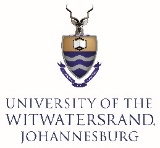


Faculty of Health Sciences · Private Bag 3, Wits, 2050, South Africa

Tel: +27 11 717 2042· Fax : +27 11 643 5415 · E-mail: ane.orchard@wits.ac.za · www.wits.ac.za

| Name and Surname: |  |
| --- | --- |
| ID number/ DOB (yyyy/mm/dd): |  |
| Signature: |  |
| Date |  |


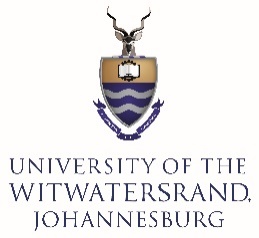


| **MANUAL ENTRY QUESTIONNAIRE FOR STEPPS WELLNESS DAY** | | | | | | | |
| --- | --- | --- | --- | --- | --- | --- | --- |
| **PERSONAL & DEMOGRAPHIC DATA** | | | | | | | |
| Date: | | | Designation: Staff Student Other | | | | |
| Please confirm that you understand and give permission for the Wellness Screenings to be performed on you and for your data to be recorded | | | - Yes - No | | | | |
| Site | | | Main Campus Education Campus Wits Res  health Sciences Campus Outside wits | | | | |
| Please indicate your age range | | | 18-24 25-34 35-44  45-54 55-64 65 and above | | | | |
| What is your sex? | | | ¨ Male ¨ Female ¨ Other I do not wish to disclose | | | | |
| Ethnicity (Self-declared) | | | ¨ African ¨ White Indian M Mixed Asian Other | | | | |
| Level of education | | | ¨ None Primary School Secondary School  Matric Certificate Certificate Diploma ¨ Bachelors/Honours Masters degree PHD | | | | |
| What is your Occupation | | |  | | | | |
| Are you on medical aid | | | ¨ Yes ¨No | | | | |
| If on medical aid, are you on any medical aid specific diabetes/hypertension/dyslipideamia program? | | | ¨ Yes No N/A | | | | |
| **MEDICAL HISTORY** | | | | | | | |
| Have you ever been diagnosed with Hypertension? (except in pregnancy) | | | | Yes No | | | |
| Do you have a family history of Hypertension? | | | | Yes No N/A | | | |
| When was your blood pressure (BP) last measured? | | | | Never Over 12 Months ago  Within the last 12 months | | | |
| Are you currently on prescribed blood pressure lowering treatment?  If yes, please specify the medication?  For how long have you been taking the medication? | | | | Yes No  Less than 1 year 1-5years >5years | | | |
| Have you ever been diagnosed with diabetes? (except in pregnancy)  If yes specify | | | | Yes No  Type 1 Type 2 Other | | | |
| Do you have a family history of diabetes? | | | | Yes No | | | |
| When was your blood glucose level last measured | | | | Never Over 12 Months ago  Within the last 12 months | | | |
| Are you currently on prescribed diabetes medication?  If yes specify the medication  For how long have you been taking the medication? | | | | Yes No  Less than 1 year 1-5years >5years | | | |
| Have you ever been diagnosed with High Cholesterol? | | | | Yes No | | | |
| Do you have a family history of high cholesterol | | | | Yes No | | | |
| When was your Cholesterol level last measured? | | | | Never Over 12 Months ago  Within the last 12 months | | | |
| Are you on any cholesterol lowering medication?  If yes specify the medication  For how long have you been taking the medication? | | | | Yes No    Less than 1 year 1-5years >5years | | | |
| Have you ever been classified as overweight or obese by a health professional? | | | | Yes No | | | |
| Are you on any prescribed weight loss medication?  If yes specify the medication  For how long have you been taking the medication? | | | | Yes No  Less than 1 year 1-5years >5years | | | |
| To your best knowledge, are you pregnant? | | | | Yes No | | | |
| How often do you consume alcohol? | | | | Never or rarely  Less than once a week  Regularly (More than one a week) | | | |
| Do you smoke? | | | | Yes No | | | |
| Are you currently fasting? | | | | Yes No | | | |
| Do you have any other chronic condition besides the ones indicated above?  If yes specify | | | | Yes No | | | |
| Are you currently taking any other chronic or natural medication?  If yes specify | | | | Yes No | | | |
| Are you currently on other medication that is not chronic?  If yes specify | | | | Yes No | | | |
| **WELLNESS TEST SELECTED (SELECT MOST APT BASED ON HISTORY)** | | | | | | | |
| - BMI 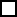 BP 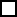 Blood Glucose 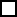 Total Cholesterol 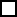 Waist Circumference 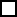 HBA1c - Lipid Panel 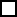 Hemoglobin 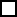 Uric acid 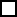 ECG | | | | | | | |
| **RESULTS** | | | | | | | |
| Weight: ______kg | | Height: ________m | | | | Waist Circumference: _______cm | |
| BP (mm/Hg) (INSERT FIGURES)  **1.** / p = | | Device used:  **2.** / p = | | | | **3.** / p = | |
| Last meal, time: | | Blood Glucose: _______mmol/l  Device: Test: Random/Fasting | | | | HbA1c:___________% | |
| Total Cholesterol: _____mmol/l  Device: | | Haemoglobin: _______mg/dl  Known anemia: Yes No | | | | Uric acid: _______mg/dl  Known gout: Yes No | |
| HIV:  +ve  -ve | Lipid Panel:  TC: _____mmol/l HDL-C: _____mmol/l Trig _____mmol/l LDL-C _____mmol/l TC/HDL Ratio: ______ | | | | | | BMI: _______kg/m^2^  ECG: _______  FRS: _______ |
| **ACTIONS TAKEN** | | | | | | | |
| Given appropriate lifestyle advice  Specify type of advice given | | | | | 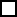 Yes 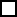No  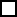Smoking 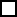 Salt restriction  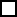 Exercise 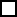 Diet  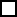Weight reduction  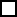Alcohol consumption | | |
| Referred for follow-up testing | | | | | 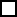Yes 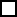 No | | |
| Referred for better management of Chronic Condition | | | | | 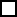 Yes 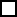 No | | |
| If referred, may we follow up on your health? | | | | | 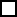 Yes 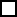 No | | |
| **STEPPS VOLUNTEER INFORMATION** | | | | | | | |
| Name | | | Date: | | | | |
| Signature: | | | | | | | |

**S 2: Follow-up survey of STEPPS CVD referred participants**

| **Follow-up survey of CVD referred participants** | |
| --- | --- |
| **Demographical data** | |
| **What is your age?** | **18-24 25-34 35-44**  **45-54 55-64**  **65 and above** |
| **What is your sex?** | **Male Female Other**  **I do not wish to disclose** |
| **Interventions post-STEPPS screening event** | |
| **Did you seek further care after being referred from the STEPPS programme?** | **Yes No** |
| **Did further care reveal anything of significance?** | **Yes No** |
|  | **If yes, severity or was it a diagnosis?**   - **Severity of the disease** - **New diagnosis: ______________** - **Other (Please specify): ____________** |
| **Did you make any lifestyle changes since the STEPPS screening event?** | **Yes No**   - **Diet** - **Exercise** - **Other (Please specify): _______________**   **If not, why____________________________________** |
| **Did you develop an established routine for screening and testing of your health condition after the STEPPS screening event?** | **Yes No**   - **Monthly** - **Every 6 months** - **Yearly**   **If not, why__________________________________________** |
| **Have you experienced any further symptoms since your last screening?** | **Yes No** |
| **What treatments are you on to manage your disease?** | - **No treatment** - **Aspirin** - **Other medicines or pills** - **Diet** - **Exercise** - **Other (Please specify): _______________** |
| **STEPPS screening event feedback** | |
| **Did STEPPS influence the intervention you chose to take?** | **Yes No** |
| **Are there any other screening tests you would like this initiative to offer?** | **Yes No** |
| **Would you like screening stations (like STEPPS) to be more accessible?** | **Yes No**  **If Yes, where? ______________________________**  **If not, why not? _____________________________** |
| **Were you comfortable during the session?** |  |
| **Were your results explained to you?** |  |
| **How likely are you to recommend these sessions to your colleagues/friends?** | **1 – Unlikely to recommend 5- Very likely to recommend**      **1 2 3 4 5** |
| **Is there any other information you would like to share?** |  |

S3: **Data analysis parameters**

***Blood pressure***

Self-reported prevalence for hypertension. The blood pressure was measured three times and an average was calculated. Anyone with an abnormal value of >140 systolic and/or >90 diastolic was referred. The referral parameters were based on the ESC guidelines as depicted in Table 1.

**Table 1: Referral parameters for blood pressure**

|  | **Classification** | **Systolic (mmHG)** |  | **Diastolic (mmHG)** |
| --- | --- | --- | --- | --- |
| **Normal** | **Optimal** | **<120** | **and** | **<80** |
|  | **Normal** | **120-129** | **and/or** | **80-84** |
|  | **High normal** | **130-139** | **and/or** | **85-89** |
| **Abnormal – Cause for referral** | **Elevated BP reading/Hypertension** | **≥140** | **and/or** | **≥90** |
|  | **Isolated systolic** | **≥140** | **and** | **<90** |
|  | **Isolated diastolic** | **<140** | **and** | **≥90** |

(1)

***Blood glucose***

Self-reported prevalence of diabetes. Any participant with abnormal values for Fasting Plasma Glucose (FPG) and Random Plasma Glucose (RPG), underwent the HbA1c testing. The clinical threshold for abnormal levels is explained in Table 2.

**Table 2: Referral parameters for glucose**

| **Blood Glucose** | **Normal** | | **Abnormal – Cause for referral** | |
| --- | --- | --- | --- | --- |
| **Fasting Plasma glucose (mmol/L)** | **<5.6**  **Diabetes excluded** | | **>5.6-6.9**  **Impaired fasting glucose** | **≥ 7.0**  **Diabetes** |
| **Random Plasma glucose (mmol/L)** | **<5.6 Diabetes excluded** | **5.6-11.0**  **Inconclusive** |  | **≥11.1**  **Diabetes** |
| **Glycated Haemoglobin (HbA1c) (%)** | **<6.5**  **Inconclusive** | |  | **≥6.5**  **Diabetes** |
| ***Table adapted from the ADA and SEMDSA guidelines (2, 3)** | | | | |

***Blood Cholesterol***

Self-reported prevalence of cholesterol. The clinical threshold for abnormal levels is explained in Table 3.

**Table 3: Referral parameters for cholesterol**

| **Blood Cholesterol** | **Normal** | **Abnormal – Cause for referral** | |
| --- | --- | --- | --- |
| **Total cholesterol (TC) (mmol/L)** | **<4.9** | **≥ 5.0** | |
| **Low-Density-Lipoprotein (LDL) Cholesterol(mmol/L)** | **<2.9** | **≥ 3.0** | |
| **High-Density-Lipoprotein (HDL) Cholesterol(mmol/L)** |  | **Men** | **<1.0** |
|  |  | **Women** | **<1.2** |
| **Triglycerides (mmol/L)** | **<1.6** | **≥ 1.7** | |
| **Table adapted from (4)** | | | |

***Anthropometric measurements***

Self-reported prevalence of obesity. The occurrence of obesity was determined by calculating the BMI using the weight (kg) and height (m) and categorizing them according to the BMI classifications guided by the WHO as shown in the Table 4. The waist circumference (WC) was used to determine the increased risk in an individual.

**Table 4: Referral parameters for BMI and Waist circumference**

| **Anthropometric measurements** | | |
| --- | --- | --- |
| **Body Mass Index (BMI)** | | |
| **Classification** | **International BMI category (kg/m^2^)** | **BMI category for Asians** |
| **Underweight** | **<18.5** | **<18.5** |
| **Normal weight** | **18.5-24.9** | **18.5-22.9** |
| **Overweight** | **25.0-29.9** | **23.0-24.9** |
| **Obese** | **≥30.0** | **≥25.0** |
| **Waist circumference (cm)** | | |
| **Men** | **≥ 94** | |
| **Women** | **≥ 80** | |

(3)

1. Williams B, Mancia G, Spiering W, Agabiti Rosei E, Azizi M, Burnier M, et al. 2018 ESC/ESH Guidelines for the management of arterial hypertension: The Task Force for the management of arterial hypertension of the European Society of Cardiology (ESC) and the European Society of Hypertension (ESH). European Heart Journal. 2018;39(33):3021-104.

2. ADA ADA. 2. Classification and diagnosis of diabetes: standards of care in diabetes. Diabetes Care. 2023;46(Supplement_1):S19-S40.

3. SEMDSA GCo. Guidelines for the Management of Type 2 diabetes mellitus. Journal OF Endocrinology, Metabolism And Diabetes Of South Africa. 2017;22(1):S1-S196.

4. Reiger S, Jardim TV, Abrahams-Gessel S, Crowther NJ, Wade A, Gomez-Olive FX, et al. Awareness, treatment, and control of dyslipidemia in rural South Africa: The HAALSI (Health and Aging in Africa: A Longitudinal Study of an INDEPTH Community in South Africa) study. PloS One. 2017;12(10):e0187347-e.
